# The best choice for low and moderate myopia patients incapable for corneal refractive surgery: implantation of a posterior chamber phakic intraocular lens

**DOI:** 10.1007/s10792-022-02459-3

**Published:** 2022-08-19

**Authors:** Qin Wang, Lina Fan, Qizhi Zhou

**Affiliations:** 1 Chongqing Aier Eye Hospital, Chongqing, 400020 China; 2 Aier Chongqing Optometry Eye hospital, No. 77, Daping Changjiang Second Road, Yuzhong, Chongqing, 400040 China

**Keywords:** Low and moderate myopia, Central hole phakic posterior chamber intraocular lens (ICLV4c) implantation, Effectiveness, Safety, Vault

## Abstract

**Purpose:**

This study aims to evaluate the early clinical outcomes of central hole phakic posterior chamber intraocular lens(ICLV4c) implantation for low and moderate myopia correction.

**Methods:**

This retrospective clinical study included 27 patients(47 eyes)who underwent ICLV4c implantation to correct myopia with spherical equivalent (SE) between − 1.75D and −6.0D. The uncorrected visual acuity (UCVA), intraocular pressure(IOP), central anterior chamber depth (ACD), vault, and endothelial cell density (ECD) were evaluated after surgery.

**Results:**

At one year follow-up, the postoperative UCVA of patients was higher than the preoperative CDVA and showed a stable trend. There was no significant difference in ECD (*p* > 0.05) one month after the surgery, the vault was 0.77 ± 0.32 mm, which decreased to 0.63 ± 0.26 mm after one year of surgery. Similarly, ACD was 3.24 ± 0.25 mm in the preoperative, which decreased significantly to 2.05 ± 0.39 mm at one month, while rose to 2.2 ± 0.39 mm at one year after surgery. There was no significant correlation between IOP and ACD and vault at one month and one year after surgery. The correlation coefficient between ACD and vault was found to be − 0.72 at one month after surgery, while the same −0.71 after one year. One patient developed visual fatigue, one with glare, and while no other complications were observed with the rest of the patients.

**Conclusion:**

The ICL implantation is a safe, effective and stable method to correct low and moderate myopia, and may be a good alternative for patients with low and moderate myopia who cannot undergo corneal refractive surgery.

## Introduction

The main surgical methods for correcting ametropia include corneal laser surgery and posterior chamber intraocular lens (ICL) implantation. Corneal laser surgery is effective and safe [1] for correcting low and moderate myopia. However, it is contraindicated for patients with a thin cornea or having potential lesions such as suspicious keratoconus [2–4]. ICL is a better option for patients not fit for corneal laser surgery since it does not involve corneal excision [5] and retains accommodation for the eyes. Additionally, it improves visual acuity compared to laser-assisted in situ keratomileusis (LASIK) [6]. Furthermore, the posterior chamber intraocular lens of astigmatism (TICL) provides greater safety and better visual acuity in the treatment of moderate and high myopic astigmatism compared to PRK [7]. Currently, ICL is the first choice for patients with high and ultra-high myopia, and is favored for patients with low and moderate myopia.

However, it also has some defects such as cataract formation, elevated IOP, TICL rotation, pigmented diffuse glaucoma, loss of endothelial cells, etc. [7]. One of the distinguishing features of ICLV4c is a hole in the center of the lens with a diameter of about 360 µm. The central hole allows the aqueous humor to flow directly into the anterior chamber through the pupil area, avoiding preoperative laser iris drilling and high intraocular pressure caused by pupil blockage. Several studies have reported its safety and effectiveness in correcting high myopia [8, 9]. However, a limited number of studies have examined the correction of low and moderate myopia by ICL. This retrospective study aimed to explore the early effectiveness, safety, and stability of ICLV4c implantation in the correction of low and moderate myopia.

## Subjects and methods

A retrospective study was conducted at Chongqing Aier Eye Hospital from January 2017 to December 2018. A total of 27 patients (47 eyes) who were treated at Chongqing Aier Eye Hospital were included in this study. The number of cases in this work is comparable to ones in previous studies [10–13] on the safety and effectiveness of ICL implantation in correcting ametropia. The criteria for ICL implantation comprised those patients whose refraction remains stable for at least one year and the annual change was within ± 0.5 D, SE was within -6.0D, the ACD ≥ 2.8 mm, the anterior chamber angle was open, ECD ≥ 2000 cells/mm^2^, patients older than 18 years old, patients without cataract, glaucoma, uveitis, retinal detachment, optic neuritis and other eye diseases, and patients without diabetes, hypertension and other systemic diseases.

The preoperative examination included: UCVA and CDVA, manifest and cycloplegic refractions, and IOP (KT-500, Kowa, Tokyo, Japan), EDC was measured using a Corneal endothelium microscope(NIDEK, Japan), The corneal topography was performed by Pentacam(OCULUS, Germany), central pachymetry (CT1000, Shin Nippon), while ultrasound biomicroscopy (MEDA Co., Ltd.) was performed to measure sulcus to sulcus diameter. The horizontal white-to-white (WTW) was obtained with electronic digital calipers under a microscope., ACD, and fundus examination were done using slit lamp microscopy. All implanted ICL V4c were produced by STAAR Company in Switzerland. ICLV4c size was calculated by using the lens calculation software (STAAR).

During all ICL implantation surgeries, a standardized procedure was followed. Three days before the surgery, one drop of levofloxacin ophthalmic preparation (Towering pharmaceutical, China) was instilled four times a day. Those who needed astigmatic intraocular lenses (TICLs) were axially labeled before surgery. Before surgery, patients were administered dilating and cycloplegic agents. The surgery was performed under surface anesthesia with promecaine hydrochloride. All surgical procedures were performed by the same experienced surgeon. An auxiliary incision of 1 mm was created, and the anterior chamber was filled with viscoelastic material. Then, the main incision of 3 mm was made. With an injector, ICLV4c was inserted into the anterior chamber, adjusted to the center, and its haptics were implanted into the ciliary sulcus. In the case of astigmatic lenses, the lens angle was marked according to the TICL surface line to make it coincide with the surface marker on the cornea. Finally, irrigation and aspiration were carried out. After surgery, the eye was coated with tobramycin dexamethasone eye ointment (S.A. Alcon Couvreur N.V.).

The patients follow-ups were made at different time intervals, including one day, one week, one month, three months and one year, to examine UCVA, CDVA, IOP, while vault, ACD and ECD were examined at one month and one year.

## Statistical analysis

Data analysis was performed with SPSS (version 22.0, IBM Corp.). For statistical analysis, visual acuity data were converted to logarithms of the minimum angle of resolution (Log MAR). Repeated measurement variance was used to analyze repeated measurement variance data. Paired data were compared by paired sample t-test. Spearman or Pearson's method was used to determine the correlation between IOP, ACD and vault. The level of significance was set at *p* < 0.05.

## Results

A total of 27 patients (47 eyes) with low and moderate myopia were enrolled in this study, aged from 19 to 37 years (7 males and 20 females), and the demographic data of the subject is listed in Table 1.There were no significant differences in patient preoperative demographic information.

**Table 1:** Preoperative demographics of the study population

Parameters	Mean ± Standard deviation
Age (y)	29.4 ± 1.67
Gender (male %)	26
SE (D)	− 4.55 ± 1.05
Spherical (D)	− 4.28 ± 1.14
Cylinder (D)	− 0.54 ± 0.67
CDVA (logMAR)	0.01 ± 0.04

CDVA: corrected distance visual acuity; SE: spherical equivalent.

**Fig. 1:**
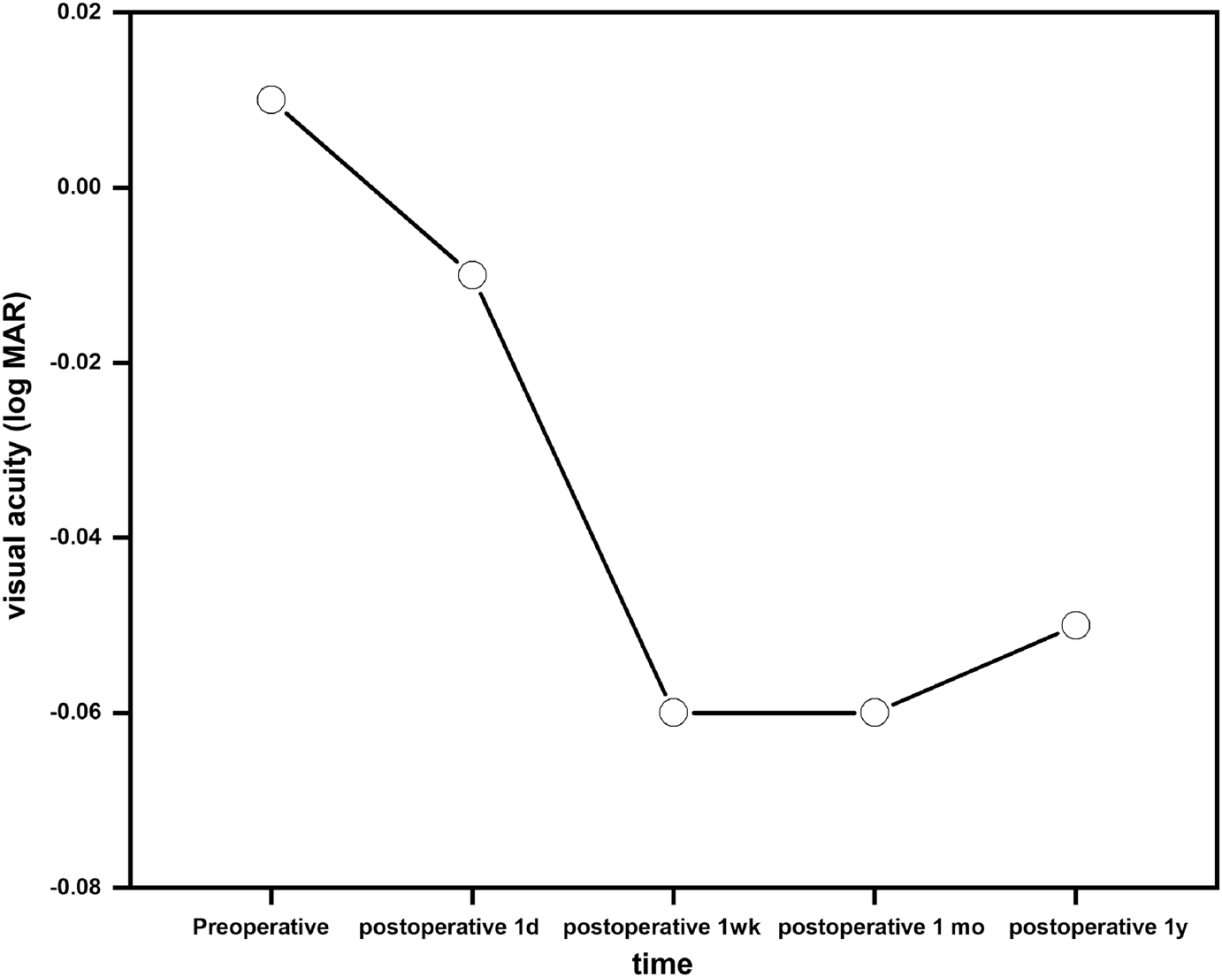
The change trend of visual acuity(Log MAR)

A comparison of preoperative and postoperative visual acuity examination results is shown in Table 2. At one-year postoperative visit, the postoperative UCVA of 75% of eyes was greater than the preoperative CDVA, and the others were equal to the preoperative CDVA. At each follow-up, visual acuity was statistically significant (F = 32.93, *p* < 0.01). The pairwise comparison revealed statistically no significant difference (*p* > 0.05) between UCVA (log MAR) one day after surgery and CDVA (log MAR) before surgery. Additionally, a statistically significant difference(*p* < 0.01) was observed when UCVA (log MAR) after surgery and preoperative CDVA (log MAR) were compared. UCVA was significantly better in patients after surgery, and this trend remained stable (Fig. 1).

**Table 2:** Comparison of preoperative CDVA (Log MAR) with postoperative UCVA (Log MAR) and preoperative and postoperative IOP ($\overline{x }$±*s*)

Parametes	Preoperative	Postoperative				*p* value
		1 d	1 wk	1 mo	1 y	
visual acuity (log MAR)	0.01 ± 0.04	− 0.01 ± 0.05	− 0.06 ± 0.06	− 0.06 ± 0.06	− 0.05 ± 0.06	< 0.01
IOP (mm Hg)	15.09 ± 2.74	13.54 ± 3.19	13.19 ± 3.3	12.31 ± 2.89	13.36 ± 2.95	< 0.001

*CDVA* corrected distance visual acuity; *UCVA *uncorrected distance visual acuity; *IOP *intraocular pressure; *log MAR *logarithm of the minimum angle of resolution

There was a significant difference in IOP before and after surgery (F = 32.7, *P* < 0.001). In pairwise comparison, there was no significant difference in IOP between one day and 1 year((*p* = 1), one month and one year after surgery (*p* = 0.098). After the first week of surgery, IOP was the lowest, and tended to stabilize after one month. When IOP before surgery was compared to IOP after surgery, a reduction of 2.8 mmHg was observed one month after surgery and 1.78 mmHg one year after operation, compared with that before operation.

There was no significant change in ECD (F = 0.152, *p* > 0.05, Table 3) at any follow-up. ACD before and after surgery at each follow-up is also shown in Table 3. In the first month after surgery, ACD decreased to its lowest level, then increased one year later. Similarly, in the first month after surgery, the vault range was 0.22 ~ 1.42 mm, with a mean of 0.77 ± 0.32 mm. After one year of surgery, the range of vault was 0.1 to 1 mm, with a mean ± standard deviation of 0.63 ± 0.26 mm. When the mean of both follow-ups was compared, a significant difference between the vault range was observed (t = 7.24, *p* < 0.05).

**Table 3:** Preoperative vs postoperative measurements($\overline{x }$ ± s)

Parameter	Preoperative	Postoperative		*p* value
		1 mo	1 y	
ECD(cells/mm^2^)	2915.45 ± 286.92	2906.03 ± 309.1	2915.49 ± 334.77*	0.85
vault (mm)	–	0.77 ± 0.32	0.63 ± 0.26†	< 0.001
ACD(mm)	3.24 ± 0.25	2.05 ± 0.39*	2.2 ± 0. 39*	*P* < 0.001

*ECD*   endothelial cell density; *ACD* central anterior chamber depth

^*^P < 0.05 (compared with preoperative value), †*P* < 0.05 (compared with 6-month postoperative value)

The correlation coefficient between vault and ACD was − 0.72 (*P* < 0.05) at 1 month after surgery and − 0.71 (*P* < 0.05) at one year after surgery. It was found that ACD was negatively correlated with vault, indicating that an increase in ACD leads to a decrease in the vault.

One patient had visual fatigue and the other had glare after surgery. However, the symptoms were significantly relieved one month later. None of patients showed signs of corneal endothelial decompensation, cataract, or glaucoma.

## Discussion

ICL implantation can preserve corneal integrity, is not limited by corneal thickness, and has a wide range of corrections of ametropia. ICL is indicated not only for patients with high and ultra-high myopia, but also for patients with low and moderate myopia with thin corneas and potential keratopathy. In recent years, ICL has become increasingly popular among ametropic patients, and its effectiveness and safety for the correction of myopia have been reported [14]. A follow-up study conducted by Kamiya et al. [15] on patients who had ICL implantation revealed that ICL can correct low, moderate and high myopia.

This study aimed to evaluate the early clinical effectiveness, safety and stability of ICL implantation in the correction of low and moderate myopia. Since ICL implantation aims to achieve high visual acuity without glasses or contact lenses, therefore, the key clinical observation index UCVA was applied in this study. The results of this study indicated that ICL is an effective correction method for patients with low and moderate myopia. Furthermore, UCVA of 94% of eyes one day after surgery was significantly higher than or equal to CDVA before surgery. There was significant difference between postoperative and preoperative visual acuity (Log MAR) (*P* < 0.01). However, there was no significant difference between the visual acuity (Log MAR) at each follow-up after surgery, indicating that visual acuity recovered quickly and remained stable after implanting ICL. These findings were consistent with previous research published on the correction of low and moderate myopia by ICL implantation [14].

After surgery, the mean IOP on the first day was found to be 13.54 ± 3.19 mmHg, whereas, after the first week, it decreased to 10.54 ± 2.9 mmHg. Similarly, after one month, a slight increase was observed and found to be 12.31 ± 2.89 mmHg, which further increased to 13.36 ± 2.95 mmHg after one year of the surgery. Statistically significant differences (*p* < 0.001) were observed when IOP scores were compared. IOP of none of patients was found abnormal. The lowest IOP was observed after the first week of surgery, which could be attributed to the effect of drugs like acetazolamide and brimonidine tartrate which were used by the patients after the surgery.

After discontinuing IOP-lowering medications, IOP reached a normal and stable state after two to three weeks. These findings were consistent with previous studies on ICL implantation[5, 16, 17].

A study by Elmohamady MN et al. [18] assessed the anterior chamber changes in 34 patients with high myopia(34 eyes) after ICL implantation. The results revealed that the angle of the chamber, the volume of the anterior chamber, and ACD decreased significantly during the first month after surgery. However, no significant differences were observed at three months, six months and 12 months postoperatively. In the present study, ACD was found to be 2.05 ± 0.39 mm at one month and 2.2 ± 0.39 mm after one year postoperatively, which was significantly less than ACD before surgery 3.24 ± 0.25 mm. The possible explanation for this could be the change of vault. Our study found an adverse correlation between vault and ACD. At one month following surgery, the vault decreased by 18.18%, compared to one year after surgery.

The vault should be monitored after the ICL has been implanted. The vault range should be 0.25 ~ 0.75 mm [19].

A lower ICL vault after implantation is associated with lens opacity [20]. A high vault can cause a shallow anterior chamber, angle closure, corneal endothelial damage, pupil occlusion or glaucoma [21]. In our study, the vault ranges from 0.22 mm to 1.42 mm one month after surgery, with an average value of 0.77 ± 0.32 mm, and it ranged from 0.17 to 1 mm one year after surgery, with an average value of 0.63 ± 0.26 mm. The vault change presents a downward trend, consistent with Schmidinger G et al.[22]. In this study, the vault was negatively correlated with ACD, with a correlation coefficient of 0.72 one month after surgery and 0.71 one year after surgery. One month after surgery, 51.06% of vaults greater than 0.75 mm and 6.4% are less than 0.25 mm. One year after surgery, 23.4% of the vaults were greater than 0.75 mm and 6.4% were less than 0.25 mm. These results indicated that the change of vault tends to a downward trend and tends to develop in the direction of safety.

Research results provided by US Food and Drug Administration revealed that the rate of ECD loss at one year, two years and three years after surgery were 3%, 5.3%, and 8.5%, respectively. In the early postoperative period, ECD loss rate was 9.9% (one month after surgery), and then decreased to 4.7% (six months after surgery). The number of ECD remained stable during the follow-up of 2 ~ 10 years [23, 24]. In our study, the preoperative ECD was (2915.45 ± 286.92) mm^2^ and the postoperative ECD was (2906.03 ± 309.1) mm^2^ at one month, which was 0.3% lower than the preoperative ECD. The postoperative ECD was (2915.49 ± 334.77) mm^2^ at one year, which was equal to before surgery.

Visual acuity of patients with low and moderate myopia improved significantly following myopia correction. However, glare persists in some patients and it is the current research hotspot. One patient had binocular fatigue in our study, and another had glare, but the symptoms were significantly relieved after a month, Other subjects did not develop any complications such as cataracts, glaucoma and corneal endothelial decompensation.

## Conclusions

Our study confirmed the effectiveness and stability of visual acuity and safety after ICLv4c implantation in patients with low and moderate myopia who are unsuitable for corneal refractive surgery. However, large sample size and longer follow-up are needed to observe its effectiveness, safety, changes in the vault, anterior lens opacification, theoretical preparation and selection of ICL size in correcting low and moderate myopia.

## Data Availability

N/A.
